# A review on invasive false indigo bush (*Amorpha fruticosa* L.): Nuisance plant with multiple benefits

**DOI:** 10.1002/ece3.9290

**Published:** 2022-09-14

**Authors:** Jasna Grabić, Branka Ljevnaić‐Mašić, Ai Zhan, Pavel Benka, Hermann Heilmeier

**Affiliations:** ^1^ Faculty of Agriculture University of Novi Sad Novi Sad Serbia; ^2^ State Key Laboratory of Soil Erosion and Dryland Farming on the Loess Plateau Northwest A&F University Yangling Shaanxi China; ^3^ TU Bergakademie Freiberg Interdisciplinary Environmental Research Centre Freiberg Germany

**Keywords:** alien species, *Amorpha fruticosa*, applied ecology, biodiversity loss, bioproducts, ecosystem management, habitat degradation, invasive plant

## Abstract

Increased mobility of people around the globe has facilitated transferring species to new environments, where some have found suitable conditions and even become invasive. False indigo‐bush (*Amorpha fruticosa* L.) is a plant native to North America but has intentionally or unintentionally spread over the Northern Hemisphere, where it often becomes invasive. The plant is especially easily dispersed within the watersheds of large rivers, where seasonal flooding is regular. Seeds and other propagules are buoyant, and when the water recedes, new plants emerge, forming dense thickets where only a few other species can co‐exist. In order to sustain native biodiversity, spread control is needed. However, mechanical control and eradication measures currently in use are labor demanding and costly, while application of herbicides is limited. On the other hand, the plant possesses a number of beneficial properties, such as phytochemical applications (medical and insecticidal effects), biocoenotic uses (honey plant, ornamental features), and ecosystem services (soil stabilization, provision of food for animals, and fiber and biomass for industry, e.g., nanocellulose). For the reasons above mentioned, the plant is considered quite controversial, and the paper discusses both aspects: potential detrimental effects when introduced to new habitats and its beneficial uses for human society. In addition, the paper presents alternative measures of spreading control (e.g., grazing) and argues that exploiting it for beneficial purposes might help spread control, thus covering the expenses of controlling its distribution.

## Graphical abstract

Amorpha fruticosa L. represents an invasive plant causing a nuisance and degrading native biodiversity on a variety of wet and mesic habitats throughout the Northern Hemisphere. On the other hand, the plant species possesses numerous beneficial applications including phytochemical and biocoenotic uses, as well as ecosystem services. Active management measures could lead to spreading control, while its exploitation for beneficial purposes might represent a viable solution ensuring long‐term sustainable ecosystem management.

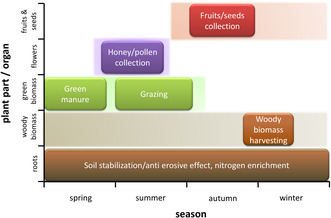

## 1 INTRODUCTION

Invasive **s**pecies are characterized by fast spreading due to producing numerous offsprings, which can be dispersed from their mother plants at large distances (Richardson et al., 2000). They have a high survival rate due to high tolerance/plasticity in response to a variety of environmental conditions. In the case of plant species and depending on the type of propagation, natural pathways can induce and accelerate spreading, especially in dramatic events such as natural disasters (floods, winds, surface erosion, etc.). Moreover, the spreading of invasive species has been significantly influenced by humans. Historically, after huge geographic discoveries, exotic plants were brought unintentionally or intentionally into new environments and cultivated as gardening plants or ornamental plants. People's increased mobility has further promoted spreading by connecting geographically distant regions (Miyawaki & Washitani, 2004) and erasing natural barriers. Subsequently, if an invasive species reaches new habitat, its ability to adapt to new conditions, coupled with biotic and abiotic factors of the host habitat, will determine its further fate in terms of survival and reproduction (Blackburn et al., 2011; Lodge, 1993). In addition, from the moment of introduction to a new environment until becoming invasive, a species has to endure and overcome different points in the invasion process defined by the unified framework proposed by Blackburn et al. (2011). Depending on the ability of a species, some were naturalized and successfully spread in the wild. Occasionally, the spreading of alien species is facilitated by coupled actions of humans and environmental properties of new habitats. There is a twofold role of human activities—transporting propagation material and altering habitats. Transport of propagation material can be intentional and unintentional. The success of invading new habitats is predominantly affected by the similarity to their natural habitats (abiotic factors), the competitive strength of native species, while anthropogenically modified habitats facilitate the spreading of nonnative species, leading to biodiversity loss and the disruption of local ecosystems and ecosystem functions (SCBD, 2006).

**FIGURE 1:**
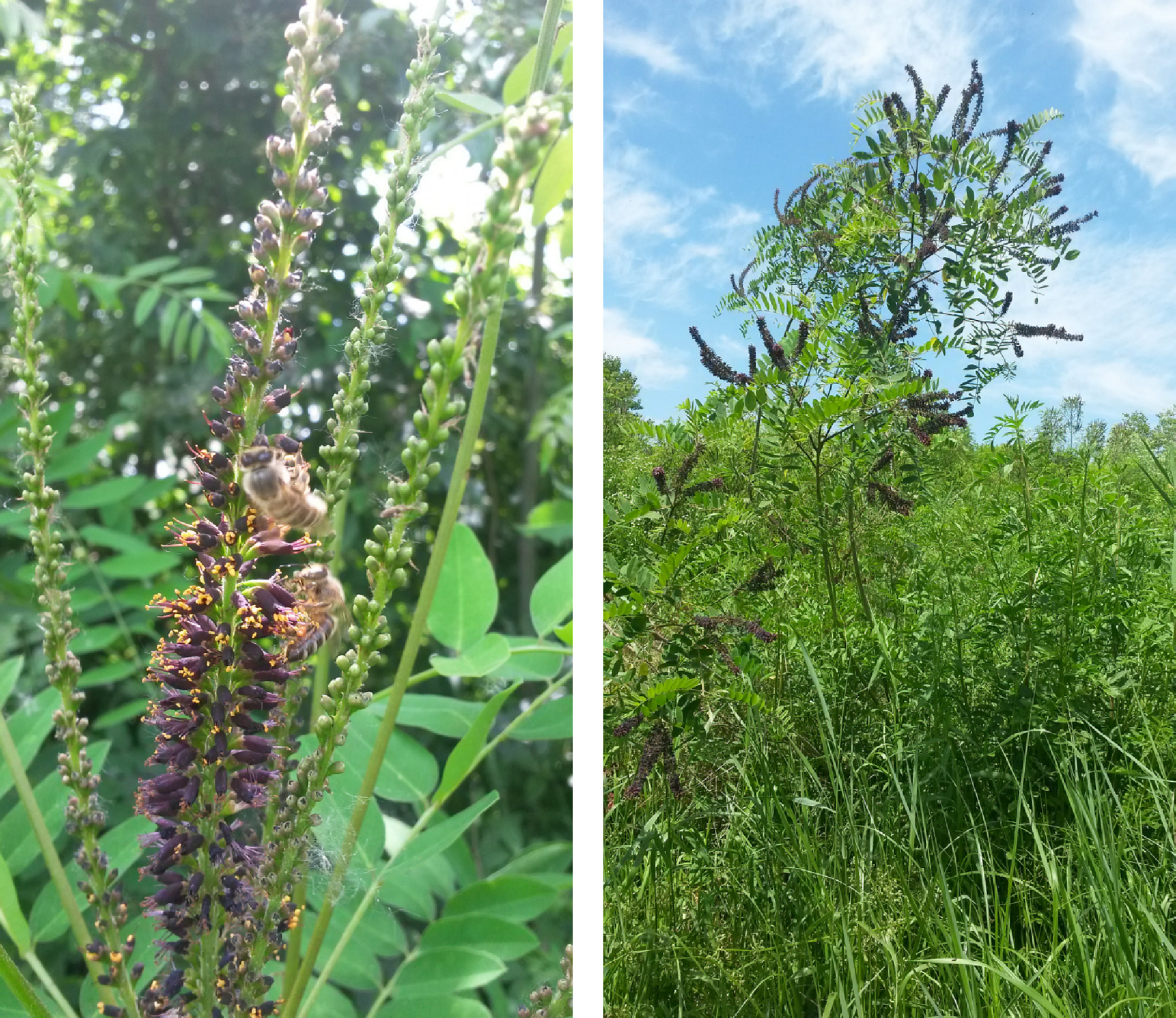
*Amorpha fruticosa* in flowering phase, Obedska Bara Special Nature Reserve, Serbia.

False indigo bush (*Amorpha fruticosa* L.), shown in Figure 1, represents a good example of an invasive plant to which all aforementioned spreading pathways can be applied. Therefore, its successful spreading from North America across most parts of the Northern Hemisphere has taken place for a few centuries. Intentional spreading of the species by humans indicates that it possesses some attributes for which it has been considered beneficial. In the case of *A. fruticosa*, there is a wide range of uses, and each organ from fruits and flowers down to its roots has some use‐value. It has potential for medicinal, food, and industrial applications (Ciuvăţ et al., 2016; DeHaan et al., 2006; Hovanet et al., 2015; Krpan et al., 2014; Zhuo et al., 2017). The above facts, that is, its invasive character and the fact that it can be exploited for various purposes, make *A. fruticosa* L. quite controversial.

Our intention is to provide an extensive overview of *A. fruticosa*'*s* history of spreading, reproductive morphology, and preferences toward the abiotic and biotic surroundings. In addition, given its controversial nature, we have examined spread control measures and listed possible uses to offer comprehensive and viable solutions for managing *A. fruticosa* in areas where it represents a threat and nuisance.

## 2 INCREASING RESEARCH ATTENTION FOR *A. FRUTICOSA*

**FIGURE 2:**
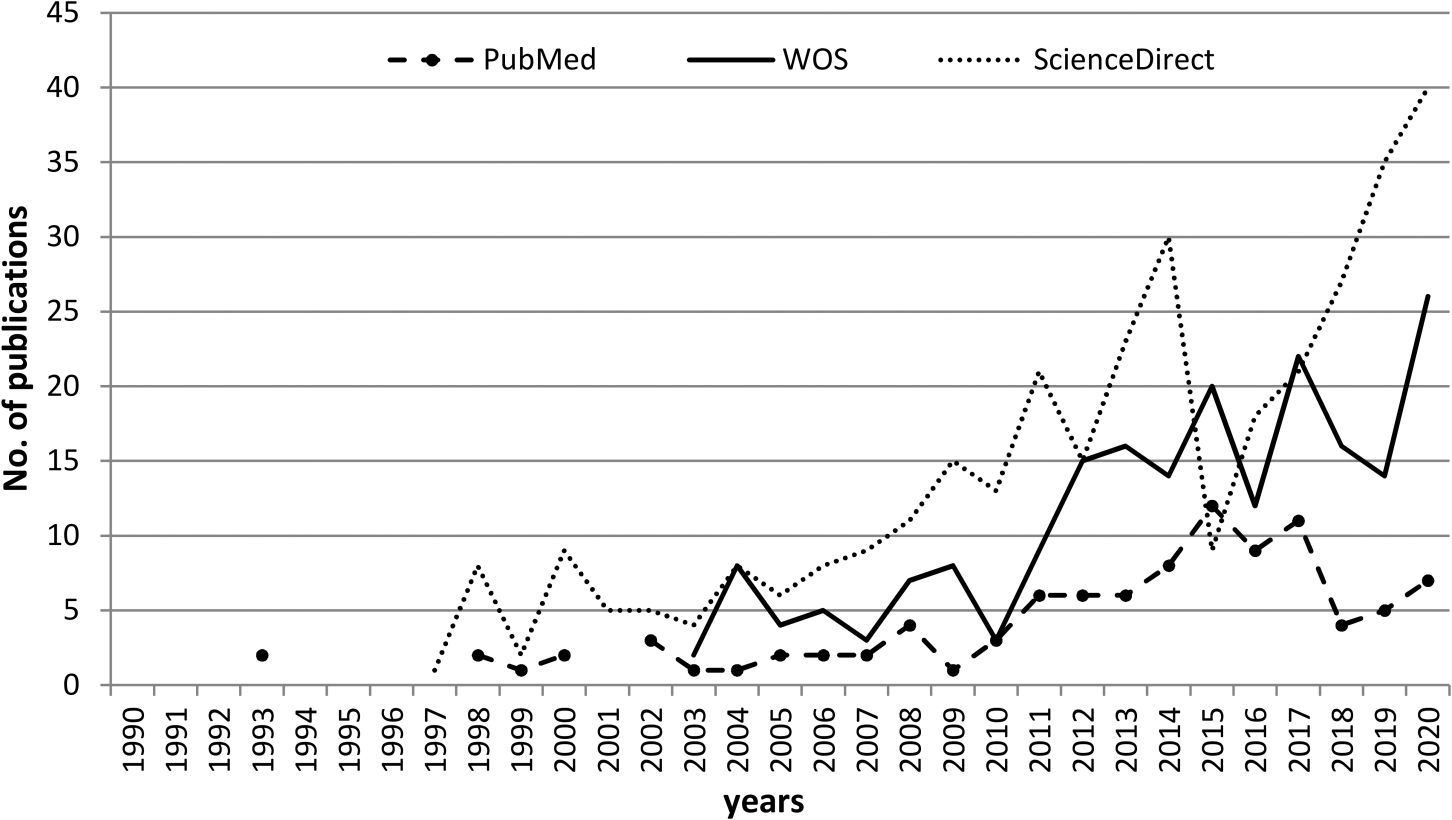
Number of publications on *Amorpha fruticosa* deposited in PubMed, WOS, and ScienceDirect, for period 1990–2020.

Data about *A. fruticosa*, concerning the species origin and distribution, habitats, and adverse and beneficial effects considering its ecology, allelopathic effects, medical, and other uses, were derived from scientific publications using services such as Web of Science—WOS, PubMed, Google Scholar, and ScienceDirect. In addition, relevant databases such as Global Biodiversity Information Facility (GBIF, 2021), Centre for Agriculture and Bioscience International (CABI, 2020), and European and Mediterranean Plant Protection Organization (EPPO) (2021) were also valuable sources of references and distribution maps. In this review, we have shown separately results for each repository. We have focused only on the number of publications during the time per repository, simultaneously avoiding debate on possible duplications, their sources, or methods for data processing. The search of major repositories and academic search engines upon using “amorpha fruticosa” as a keyword revealed the following numbers of publications: ScienceDirect—417, Scopus—369, WOS—226, PubMed—97 and Google Scholar around 13.700. There is an evident increase in publication number in ScienceDirect, followed by WOS and PubMed, particularly in the second decade of the 21st century after being at a low level of <10 publications per year since the 1990s, indicating that it is a vivid field of investigation (Figure 2).

**FIGURE 3:**
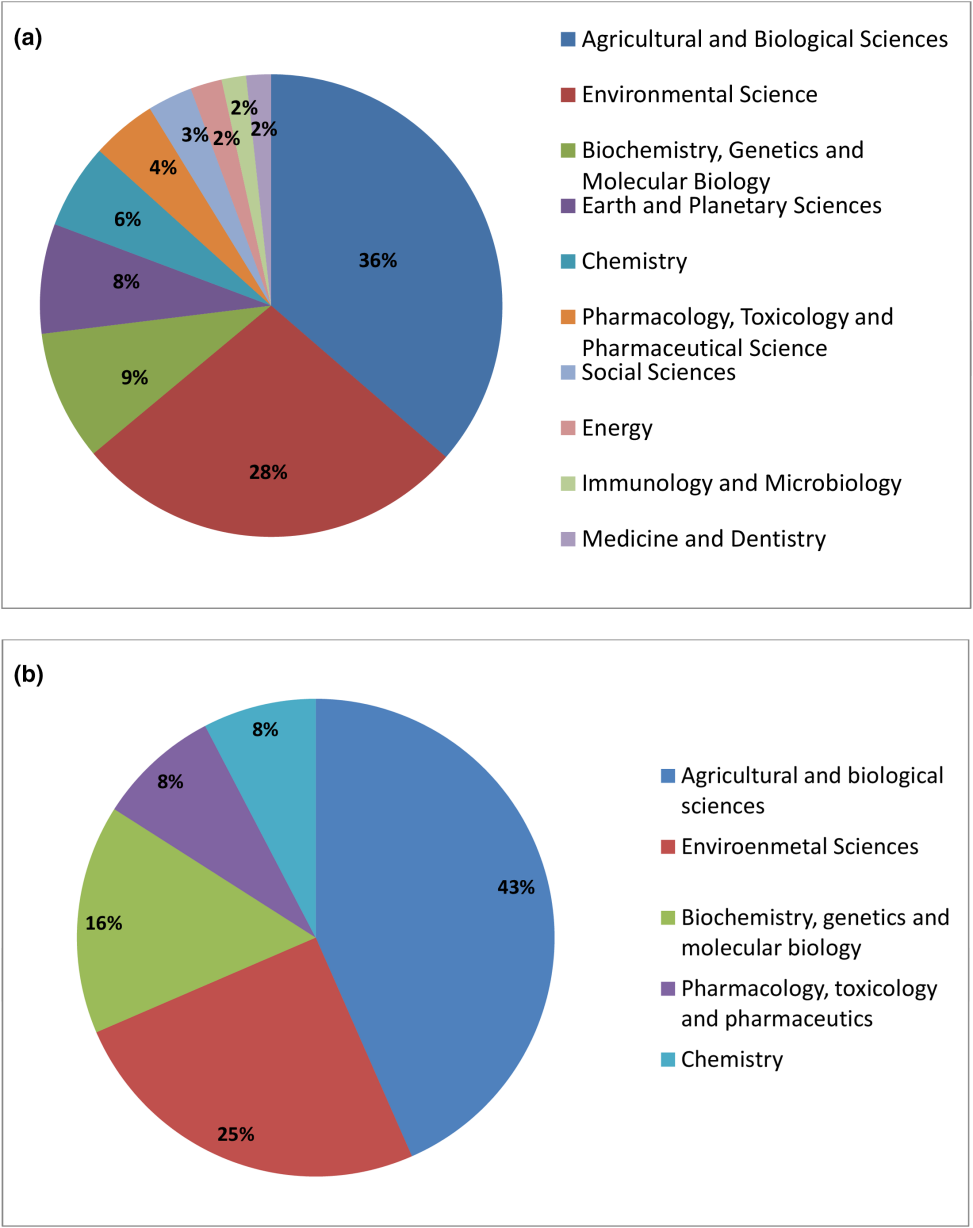
Representation of publications on *Amorpha fruticosa* concerning research area according to: (a) ScienceDirect and (b) Scopus.

Moreover, our goal was to emphasize fields in which most publications were published concerning *A. fruticosa*, that is, to identify research fields which are in the focus of the scientific community. Fortunately, ScienceDirect and Scopus provided such a possibility automatically. Therefore, we are presenting results just for these repositories. According to ScienceDirect, the most represented are publications related to agricultural and biological sciences (36%) and environmental sciences (28%), whereas for the same areas by Scopus, the share is 43% and 25%, respectively (Figure 3). In addition, publications in other categories are represented by less than 10%, for example, in biochemistry, genetics and molecular biology; Earth and planetary sciences, as well as in chemistry; pharmacology, toxicology, and pharmaceutics; social sciences; energy (Figure 3a). Similarly, by Scopus, publications related to biochemistry, genetics and molecular biology (16%), pharmacology, toxicology and pharmaceutics (8%), and chemistry (8%) are less represented (Figure 3b).

The general conclusion that can be drawn from the representation of publications by research fields (Figure 2) implies that investigating *A. fruticosa* from the ecological and environmental perspective presently dominates over research related to its molecular and phytochemical nature. Therefore, this paper provides a comprehensive review encompassing *A. fruticosa* distribution, biology, and ecology, including invasiveness and control/management. Nevertheless, beneficial uses are also included since its valuation may help in spread control.

## 3 ORIGIN AND DISTRIBUTION

**FIGURE 4:**
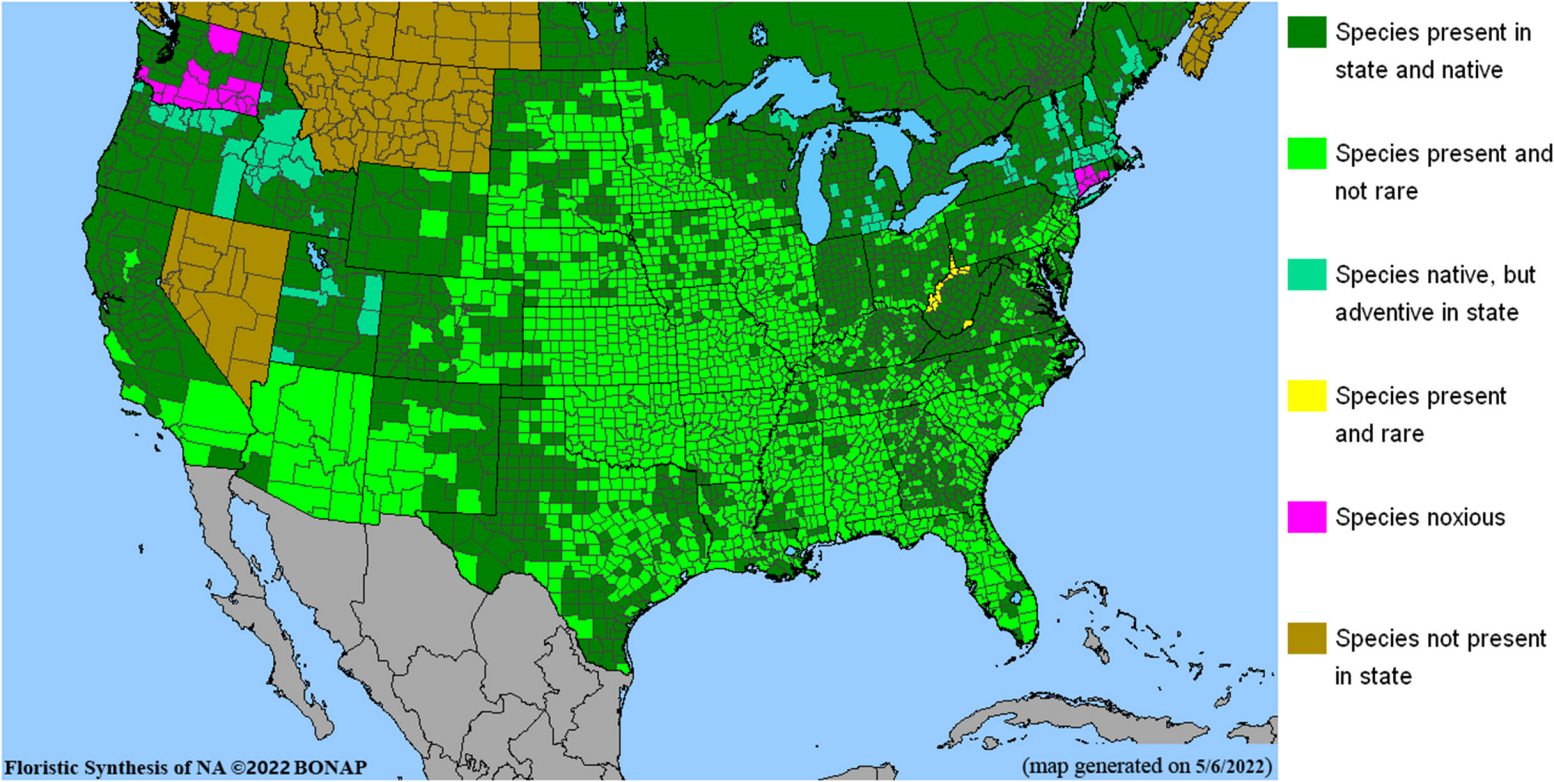
Native distribution of *Amorpha fruticosa* and its present range within the North American continent.

*Amorpha fruticosa* is native to North America. Its native range extends from southern Canada to northern Mexico, west to California, and east to Florida (Gleason & Cronquist, 1991; Ulrich & Zaspel, 2000). In several states of the United States, it is regarded as a noxious weed (DiTomaso et al., 2013; Glad & Halse, 1992). Native range and its present distribution are shown in map (Figure 4).

The first records on its introduction to Europe date back to 1724 when it was brought as an ornamental plant to England (Karmyzova, 2014). Afterward, it used to be widely planted in Europe at the beginning of the 20th century and was introduced in North Asia before the middle of the same century (Jung, 2014; Takagi & Hioki, 2013; Ulrich & Zaspel, 2000; USDA‐ARS, 2021). Presently *A. fruticosa* is reported to be invasive in a number of European countries (EPPO, 2021; Roy et al., 2020). In Europe, it has been cultivated for its ornamental features (Cullen, 1995) and as a honey plant. In addition, due to its protective properties against soil erosion, it has been intentionally dispersed along freshly built canals to stabilize embankments, especially in some regions of southeast Europe, where later it has become naturalized. The first written data on the presence of *A. fruticosa* in southeast Europe, for example, in Hungary and Bulgaria, dates back to the 1920s and 1930s (Pedashenko et al., 2012; Szentesi, 1999; Szigetvári & Toth, 2008). Presently in this region, it has been recognized among the most invasive species, and spread control is urgently needed (Doroftei et al., 2005; Gudžinskas & Žalneravičius, 2015; Körmöczi, 2012; Kozuharova et al., 2017; Kucsicsa et al., 2018). Gudžinskas and Žalneravičius (2015) reported that *A. fruticosa* was first found in 2015 in Lithuania as naturalized and potentially invasive, and the same can be assumed for Central Russia (EPPO, 2021). In addition, in the south of the Russian Far East, it has been present in botanical gardens and landscape design of urban and suburban areas (Kolyada & Kolyada, 2018). In China, *A. fruticosa* is a common shrub of its temperate regions widely planted as a windbreaker (Liu et al., 2005) and intentionally spread on Loess Plateau to stabilize soil (Yan et al., 2017) across the Delta of the Yellow River (Guo et al., 2018). In Japan, *A. fruticosa* was introduced from the eastern part of North and Central America to revegetate artificial slopes. However, it has later spread out to watersheds of rivers Hokkaido, Honshu, Shikoku, Kyusyu, and Okinawa (Hioki et al., 2015), posing now a significant threat to the local biodiversity. In the future, its ornamental value might be the reason for further spreading to the rest of Asia and also potentially to other continents, such as Africa and Central America (CABI, 2020).

**FIGURE 5:**
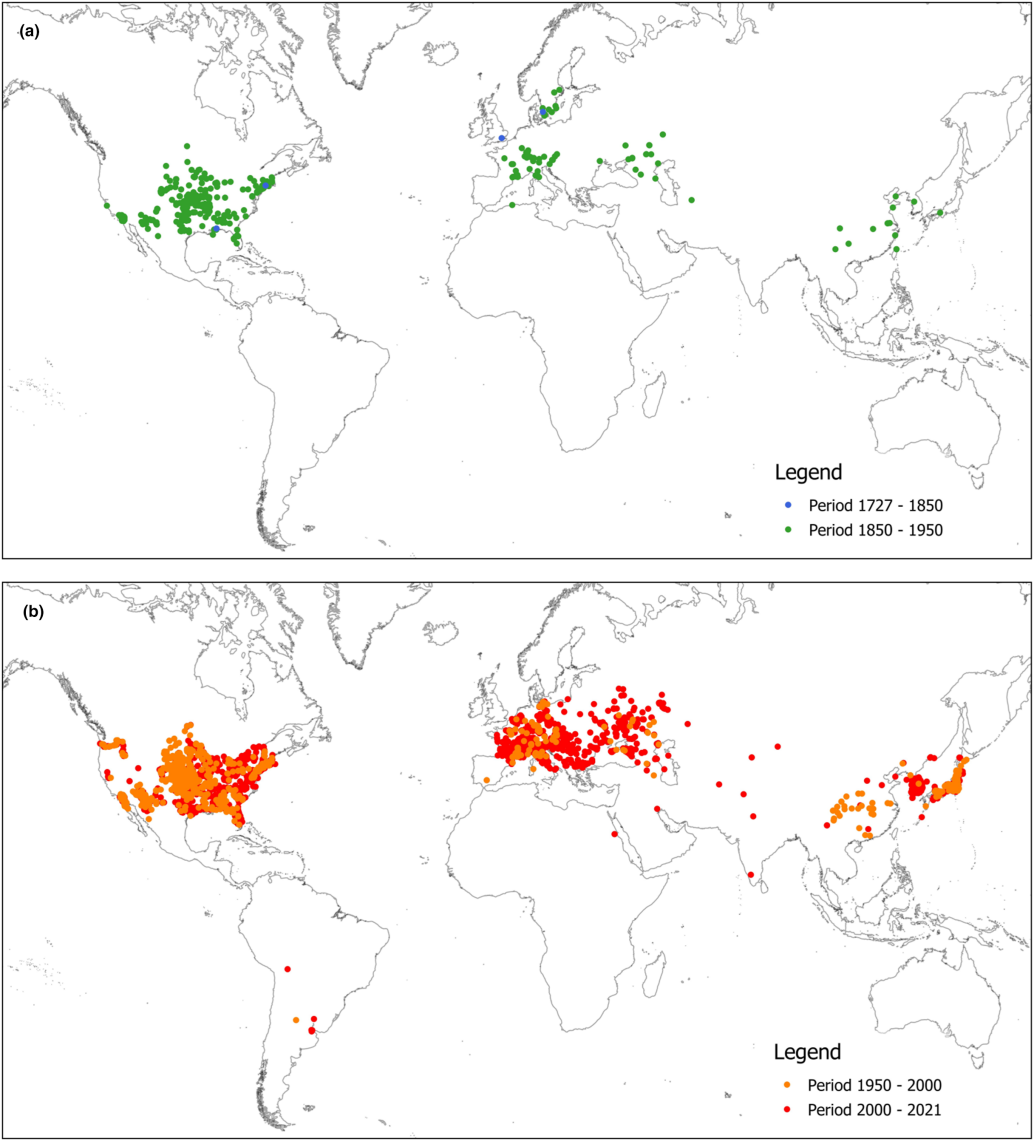
Distribution map of *Amorpha fruticosa*: (a) for period 1727–1950 and (b) for period 1950–2021 (GBIF, 2021).

Global databases such as EPPO, CABI, and GBIF can give insight into present distribution, but also the history of spreading. For example, the GBIF database presently stores more than 16,000 records, of which for more than 8000, the exact location is provided, accompanied by the date of observation. Using geo‐positioned data, we have produced maps showing the distribution of *A. fruticosa* for certain periods from its first records until the present day (Figure 5). It is evident from the map (Figure 5a) that in the second half of the 19th and the first half of the 20th century, the species has been brought to new habitats considerably remote from its native ones. This includes Europe, Asia, and the Far East, but also spreading across the North American continent. And concerning its native range, there is an obvious lack of georeferenced data for *A. fruticosa* for the period 1727–1850. The second map (Figure 5b) shows the species distribution for 1950–2000 and 2000–2021 periods, where the increased density of records in regions where it was found before 1950, but also a few new records in Central Asia and a few in South America can be noticed.

## 4 BIOLOGY AND ECOLOGY

### 4.1 Reproductive biology

*Amorpha fruticosa* is a fast‐growing shrub, which reproduces sexually—by producing a large number of seeds. Pollination is performed by insects, mainly bees, belonging to the genus *Andrena* (CABI, 2020; Halbritter & Heigl, 2021), and also by *Apis mellifera* L. Pollen is small (10–25 μm), isopolar, oblate, with three colporous aperture (CABI, 2020; Halbritter & Heigl, 2021). Apart from sexual reproduction, it can also proliferate vegetatively (asexuate) by sprouting, and stems can root at the nodes (Szigetvári, 2002), generating spindles from its superficial roots. These spindles can be very well‐developed and ramify widely (Harold et al., 2005). In response to flooding events, *A. fruticosa* forms adventitious roots (Kozlowski, 1997). It is considered to be a facultative halophyte and tolerates medium saline soils, since germination is inhibited at 3000 mg/L NaCl, while reduced values of germination parameters were recorded at concentrations of 700 and 1400 mg/L of NaCl (Đukić et al., 2010). One of the reasons might be that rhizobial strains isolated from *A. fruticosa* were not tolerant to salt concentrations above 1% NaCl (Ulrich & Zaspel, 2000).

In alluvial soils near rivers, almost 2/3 of the species' seeds stay in the upper soil layer up to 10 cm, while almost 1/3 can be found in the 10–20 cm soil layer (Blagojević et al., 2015). The same research revealed that a soil layer of 0–30 cm contained 3270 seeds/m^2^, with a germination percentage of 3.73%, that is, the number of potential plants was 122 plants/m^2^. The spreading of *A. fruticosa* is facilitated by its seed pods being buoyant and spreadable by water (Blagojević et al., 2015; Szigetvári, 2002). In addition, birds and small mammals are also reported to feed on seeds, for example, specimens of *Parus* sp. consume *A. fruticosa* seeds, which might also help the species' propagation (Doroftei et al., 2005).

### 4.2 Habitat

*Amorpha fruticosa* grows in a wide range of habitats, including riparian and alluvial habitats, sandy banks of ravines, coastal areas, dunes, and disturbed land, such as plantations, orchards, meadows, urban areas, and fishing pond depressions (Botta‐Dukát, 2008; Doroftei et al., 2005; Dumitrascu et al., 2013; EPPO, 2021; Karmyzova, 2014; Szigetvári, 2002). It often can be found on wet habitats dominated by *Salix alba* and *Populus alba* galleries, riparian galleries and thickets, alluvial forests with, riparian mixed forests, and along the great rivers (Dumitrascu et al., 2013; EPPO, 2021). The species can be rarely found on the edges of water bodies that are constantly wet (Pedashenko et al., 2012), since it does not tolerate constantly wet conditions, but only temporary during flooding periods. It is taught to be weak competitor in forests, usually suppressed by tree species (Szigetvári, 2002). However, according to our observations in Special Nature Reserve Obedska Bara, Serbia, it dominates bush layer in mixed forest of English oak (*Quercus robur* L.), manna ash (*Fraxinus ornus* L.), black poplar (*Populus nigra* L.), and common hornbeam (*Carpinus betulus* L.), whereas on meadows and pastures, it absolutely predominates in a few years. It succeeds thanks to a number of its attributes, that is, fast growth, shading competitors, its nitrogen‐fixing ability (Boscutti et al., 2020), and suppressing allelopathic effects (Csiszár, 2009; Xiao et al., 2016). Its ability to inhibit the germination and growth of other plant species by the release of allelopathic substances was confirmed by Csiszár (2009 ) and Csiszár et al. (2013) who found out that the juglone index for *A. fruticosa* was near 1 for lower extract concentration or 2 for higher extract concentration, while Xiao et al. (2016) proved inhibition in growth of some medical plants planted in the humus soil of *A. fruticosa*. This may suggest that allelopathic effects could contribute to the overall success of the invasion process. Expansion of *A. fruticosa* not only contributes to biodiversity decrease but might also lead to the formation of almost impenetrable stands together with other vine or shrub species (e.g., *Echinocystis lobata*, *Robinia pseudoacacia*, *Prunus spinosa*, *Rosa canina*, and *Rubus* sp.) (Glišić et al., 2014; Sándor & Kiss, 2008).

The species prefers a warm temperate climate with dry summer or dry winter, or wet all year, and continental climate with dry summer, or wet all year (CABI, 2020) (Table 1). Concerning low temperatures, it seems that the number of frosty days influences seed germination (Doroftei et al., 2005). *Amorpha fruticosa* inhabits soils of acid, alkaline or neutral chemical reaction, and light or medium soils texture (CABI, 2020). It grows in well‐drained soils, medium to wet. Although it prefers to grow along watercourses, it can tolerate dry soils and occasional flooding. Its well‐developed root system enables it to be relatively wind‐tolerant (Doroftei et al., 2005; Kozuharova et al., 2017). The species prefers to grow at sites with high illumination (Takagi & Hioki, 2013), but it also tolerates partial shade (Doroftei et al., 2005). Regarding pH, *A. fruticosa* studied in the Danube Delta was tolerant to a pH range of 5.8–7.6 (Doroftei et al., 2005), while according to other sources, the range it tolerates is wider, that is, 5.0–8.5 (Harold et al., 2005; USDA, 2019).

**TABLE 1:** Climate that *A. fruticosa* L. prefers (CABI, 2020; Peel et al., 2007)

Climate^a^	Criteria
**Cs**—Mediterranean climate ‐warm temperate climate with dry summer	Warm average temp. >10°C, cold average temp. >0°C
**Cw**—Dry‐winter subtropical climate; warm temperate climate with dry winter	Warm average temp. >10°C, cold average temp. >0°C
**Cf**—Warm temperate climate, wet all year	Warm average temp. >10°C, cold average temp. >0°C
**Ds**—Continental climate with dry summer	Warm average temp. >10°C, coldest month <0°C
**Df**—Continental climate, wet all year	Warm average temp. >10°C, coldest month <0°C

a According to Köppen‐Geiger climate classification (Peel et al., 2007).

### 4.3 Ecology

Belonging to the order Fabales, *A. fruticosa* establishes mutual relationships with symbiotic bacteria, which enable capturing and binding atmospheric nitrogen, thus promoting plant growth and contributing to soil fertility. Research on rhizobial strains nodulating *A. fruticosa* compared to other legumes confirmed that *A. fruticosa* as a neophytic plant could form nodules with several phylogenetically different rhizobia. This might be an important attribute for adapting to new habitats compared to archaeophytic plants, which are specialized and host only one or a few specific microsymbionts (Ulrich & Zaspel, 2000).

Branches and leaves of *A. fruticosa* are dense, clustered, fast‐growing, and closed early, leading to relatively fast ground covering (Yin, 1993). This characteristic can be assumed as positive from the aspect of *A. fruticosa* since the plants get relatively resistant to environmental conditions. However, from the point of view of other plant species forming native vegetation, dense growth, and abundant shade inhibit the growth of other native, where especially herbaceous plants are susceptible. It provides food not only to bees but also to some other insects such as *Zerene cesonia* (CABI, 2020). Moreover, a few bird species of order *Passeriformes* were found within a canopy of *A. fruticosa* (Doroftei et al., 2005).

The species well tolerates waterlogged stress (Wang et al., 2012) and can grow in temporary wet conditions. It is considered to be facultative halophyte, since germination is inhibited at 3000 mg/L of NaCl, while reduced values of germination parameters were recorded at concentrations of 700 and 1400 mg/L of NaCl (Đukić et al., 2010).

## 5 INVASIVENESS OF *A. FRUTICOSA*

It seems that *A. fruticosa* does not represent a threat in terms of invasiveness within its natural habitats. For example, according to Hupp and Osterkamp (1996), *A. fruticosa* contributes to forming riparian vegetation of the Plum Creek, Colorado (USA). There, it has been listed in 6th place (out of 8), by importance, of common woody species. However, it has been introduced in to the states of New England and Washington, but it was recognized as a noxious weed, that is, Connecticut (USDA, 2019) and Washington (WS NWCB, 2022).

Concerning invasiveness, *A. fruticosa* is now generally recognized as one of Europe's most invasive alien species (Figure 5). It has a high reproductive capacity, forms dense thickets and outcompetes native flora, changing successional patterns, and reducing biodiversity (CABI, 2020; Cronk & Fuller, 2001; Glišić et al., 2014). The main natural factor contributing to invasions is flooding which facilitates the dispersal of seeds and other propagation material by water (Pyšek & Prach, 1994). Another argument is the fact that river corridors are characterized by longitudinal continuity, as recognized in the river continuum concept (Rood et al., 2010). Therefore, habitats with wet and mesic conditions are more susceptible to invasions than dry ones (Botta‐Dukát, 2008). In addition, the Danube watershed's dense hydrological network and favorable continental climate facilitated the spreading of its water‐dispersed propagules (Pedashenko et al., 2012; Pyšek & Prach, 1994).

**FIGURE 6:**
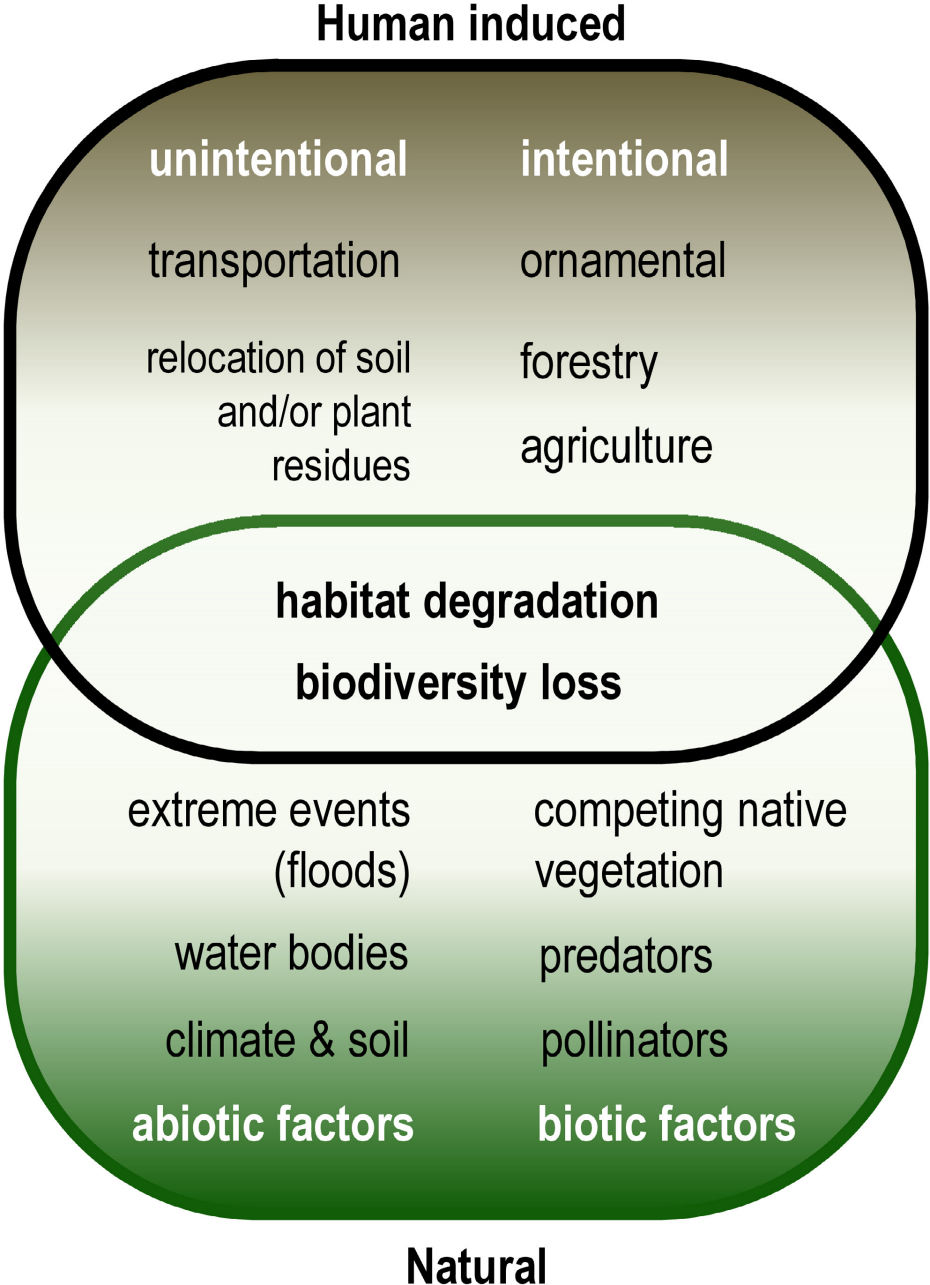
The main natural and human induced factors responsible for the introduction and spreading of *Amorpha fruticosa* and final outcomes (within the intersection).

In Europe, during the past two decades, a significant presence of *A. fruticosa* causing nuisance has been reported in Hungary, Bulgaria, and Romania (Kucsicsa et al., 2013; Szigetvári, 2002). In other southeast European countries, for example, Croatia, Slovenia, and Serbia the species is recognized as highly invasive within the Sava River Basin (Blagojević et al., 2015; Kus Veenvliet, 2021). The main factors responsible for the introduction and spreading of *A. fruticosa* are presented in Figure 6. Generally, it can be concluded that *A. fruticosa* is an important invasive species in Europe (CABI, 2020; Roy et al., 2020) and Asia (CABI, 2020).

### 5.1 Impact on habitats and biodiversity

A nonnative species in new forest habitats may have profound and cascading effects, reflected in various aspects, starting from modifying tree species composition to changes in nutrient, carbon, and water cycle (Boscutti et al., 2020; Liebhold et al., 2017; Pellegrini et al., 2021). Additionally, *A. fruticosa* has especially a pronounced impact in soil enrichment by N due to nitrogen‐fixing ability. The mentioned property causes alterations in the N cycle, which leads to cascading effect on other soil functions, eventually decreasing the biodiversity of native vegetation (Boscutti et al., 2020). This assumption can be applied to *A. fruticosa*, which is considered not just as invasive species, but rather a transformer species that invades disturbed areas (Kozuharova et al., 2017; Protopopova et al., 2006; Szigetvári, 2002). Due to its nitrogen‐fixing ability, *A. fruticosa* enriches the soil with nitrogen and substantially changes its content, thus making less favorable conditions for native flora. Therefore, it is characterized as a transformer species (Pellegrini et al., 2021). In addition, its outstanding characteristics, such as rapid growth, fast closing, and formation of dense thickets, contribute to its ability to outcompete native flora. All the mentioned properties lead to native habitat fragmentation and loss, deteriorating ecosystem structure and functioning, changing successional patterns, and finally reflecting on overall biodiversity decrease (Cronk & Fuller, 2001; De Poorter et al., 2007; Kucsicsa et al., 2018; Sărăţeanu, 2010). Despite the mentioned impacts, not all regions and vegetation types are affected by the same intensity (Vitousek et al., 1997). For example, the most susceptible to invasions are floodplain habitats, where dense stands of *A. fruticosa* prevent flood conveyance, thus disturbing natural dynamics of floodplain ecosystems (Kiss et al., 2019; Nagy et al., 2018), together with already mentioned negative allelopathic effects of *A. fruticosa* (Csiszár, 2009). In addition, habitat alterations made by *A. fruticosa* overgrowth significantly influence the composition of soil invertebrates, which are not directly related to the plant but could be instead attributed to microclimatic conditions of changed habitats (Brigić et al., 2014). Namely, while open habitat carabid beetle species declined, eutopic carabids positively reacted to the invasion in terms of increased abundance and mean individual biomass, that is, increased occurrence of larger individuals. Nevertheless, there are some positive effects, for example, *A. fruticosa* is a host plant for the planthopper *Acanalonia conica* (EPPO, 2021) and numerous insects pollinators benefit from its flowers.

## 6 PREVENTION AND SPREADING CONTROL (BIOLOGICAL, CHEMICAL, AND MECHANICAL)

In its native habitats in North America *A. fruticosa*, has a parasite. It is bruchid seed‐beetle Acanthoscelides pallidipennis (Motschulsky, 1874) that feeds on *A. fruticosa* seeds. However, in new habitats, *A. fruticosa* does not have parasites. On some occasions, together with the introduction of *A. fruticosa*, it has been followed by the introduction of the predator beetle, for example, in Japan and in the Russian Far East (Kuprin et al., 2018; Tuda et al., 2001). Finally, research carried out by Gagić‐Serdar et al. (2013) proved the potential of *A. pallidipennis* as a biological control agent of *A. fruticosa*.

Across invaded areas, *A. fruticosa* has been controlled in many ways, including mechanical, chemical, and biological control. The most frequent way of spread control is mechanical by cutting. Therefore, repeated cutting and mowing have been reported as a successful method for controlling populations in disturbed habitats (CABI, 2020). Takagi and Hioki (2013) observed that trumping and leaving plants in autumn in the vicinity of a riverbed is not a successful management strategy. Additionally, some herbicides have also proven to be successful in spreading control (CABI, 2020). Namely, glyphosate and triclopyr trimethylamine have been proven to successfully suppress *A. fruticosa* in disturbed habitats in Serbia (Blagojević et al., 2015). Burning as a natural method has also been tested, and *A. fruticosa* showed a certain resistance to fire regime (Doroftei et al., 2005; Gregory & James, 2003; USDA, 2019). Doroftei et al. (2005) have conducted different experiments like stem planting, burning, cutting, or pulling out juvenile plants. The observations show that all the tested plants developed a few new spindles after burning and many spindles after cutting the following year. The planting experiment of the cut‐away stems shows that *A. fruticosa* developed new roots and sprouts (Doroftei et al., 2005). Szigetvári (2002) and Demeter et al. (2021) demonstrated that the best results of control in floodplain meadows and poplar plantations affected by *A. fruticosa* are achieved by applying continuous moderate or intensive cattle grazing. The plant is considered unpalatable for most invertebrates except for *A. pallidipennis* seed predators, but ruminants feed on its leaves and young shoots (Szigetvári, 2002). Mechanical control is the primary means of control within protected areas since the application of chemicals is prohibited (Ciuvăţ et al., 2016). In man‐made habitats such as in poplar plantations, agrochemical measures are permitted. Regular management usually assumes replanting, but with previous soil plowing and removing old logs. During the procedure, root fragmentation occurs, which contributes to plant survival and propagation (Pedashenko et al., 2012). In such cases, if eradication is the goal, chemical control must be employed.

Frequently, where spread control/eradication of *A. fruticosa* is done in a watershed, there is a risk of permanent supply of propagation material due to the yearly dynamic of flooding. In addition, in circumstances where *A. fruticosa* transforms soil the best results, concerning its control and revegetation with native vegetation, could be achieved until overgrowth with *A. fruticosa* reaches an intermediate stage since native vegetation can still develop undisturbed (Pellegrini et al., 2021). After applying measures for *A. fruticosa* control, it is necessary to strengthen natural communities by colonizing native species (Demeter et al., 2021; Szigetvári, 2002). In addition, to keep achieved results sustainable, it is necessary to constantly mow twice a year, practice continuous grazing, or apply other control measures. Especially, the best results of control in floodplain meadows and poplar plantations affected by *A. fruticosa* are achieved by applying continuous moderate or intensive cattle grazing (Demeter et al., 2021; Kus Veenvliet, 2021). Such efforts are essential in preventing seeds setting and plants regeneration from sprouts (Szigetvári, 2002). Finally, to successfully combat the spreading of *A. fruticosa*, transnational actions are necessary, such as the SAVA Ties project. The project has summed up and tested the best strategies for several invasive species within the investigated area in terms of spreading control and management and again concerning *A. fruticosa* regular mowing and continuous grazing has proven to be the most effective long‐term measure (Kus Veenvliet, 2021).

## 7 FUTURE PROSPECTS IN MANAGEMENT OF *A. FRUTICOSA*

Although a number of methods have been tested for *A. fruticosa* spread control with certain efficiency, many authors agree that placing a value on the species (Ciuvăţ et al., 2016; Kozuharova et al., 2017), might increase motivation of area managers and can contribute to both aspects: its beneficial applications and spread control on invaded habitats. Namely, obtaining a variety of valuable and costly processed products from *A. fruticosa*, which could be used as a base raw material (e.g., for pharmaceutical purposes, nanocellulose) can help cover relatively expensive mechanical and/or labor‐intensive operations in collecting plant material. This strategy is especially recommendable in protected areas in alluvial areas, where significant efforts have to be made to control its spreading, since regular annual flooding keeps bringing new propagation material. Therefore, active management measures should be in place, focused on promoting native vegetation, while simultaneously controlling this invasive plant. Within protected areas, where direct chemical pollution is limited only to sources outside protected areas, even invasive plants could be exploited for pharmaceutical purposes, thus representing unique ecological service and sustainable development strategy (Kozuharova et al., 2017).

Another side of the problem lies in changes in land use. Abandoning traditional extensive human disturbance regime, such as mowing and grazing, pastures and meadows have transformed into gallery forests due to natural succession. The absence of these actions contributes to and facilitates *A. fruticosa* colonizing the meadow communities, leading to the formation of dense homogenous thickets within 5–6 years (Kóra, 2002), or even faster 4–5 years (Pellegrini et al., 2021). This process results in a significant biodiversity decrease, where only rare meadow plant species can survive and compete under the closed canopy of *A. fruticosa* (Kóra, 2002; Zavagno & D'Auria, 2001).

As a precaution, if *A. fruticosa* is intentionally introduced for commercial or environmental purposes, control measures must be first tested on a pilot area to select the best measure before planting on vast areas (Hioki et al., 2015). Bearing in mind that *A. fruticosa* is mainly spread by water; it can be assumed that dams and reservoirs may prevent the downstream spreading of an invasive species. Furthermore, such man‐made structures lead to fragmentation of the river corridor and impede the transfer of invasive species propagules, thus providing an environmental benefit (Rood et al., 2010).

### 7.1 Beneficial uses of *A. fruticosa*

**FIGURE 7:**
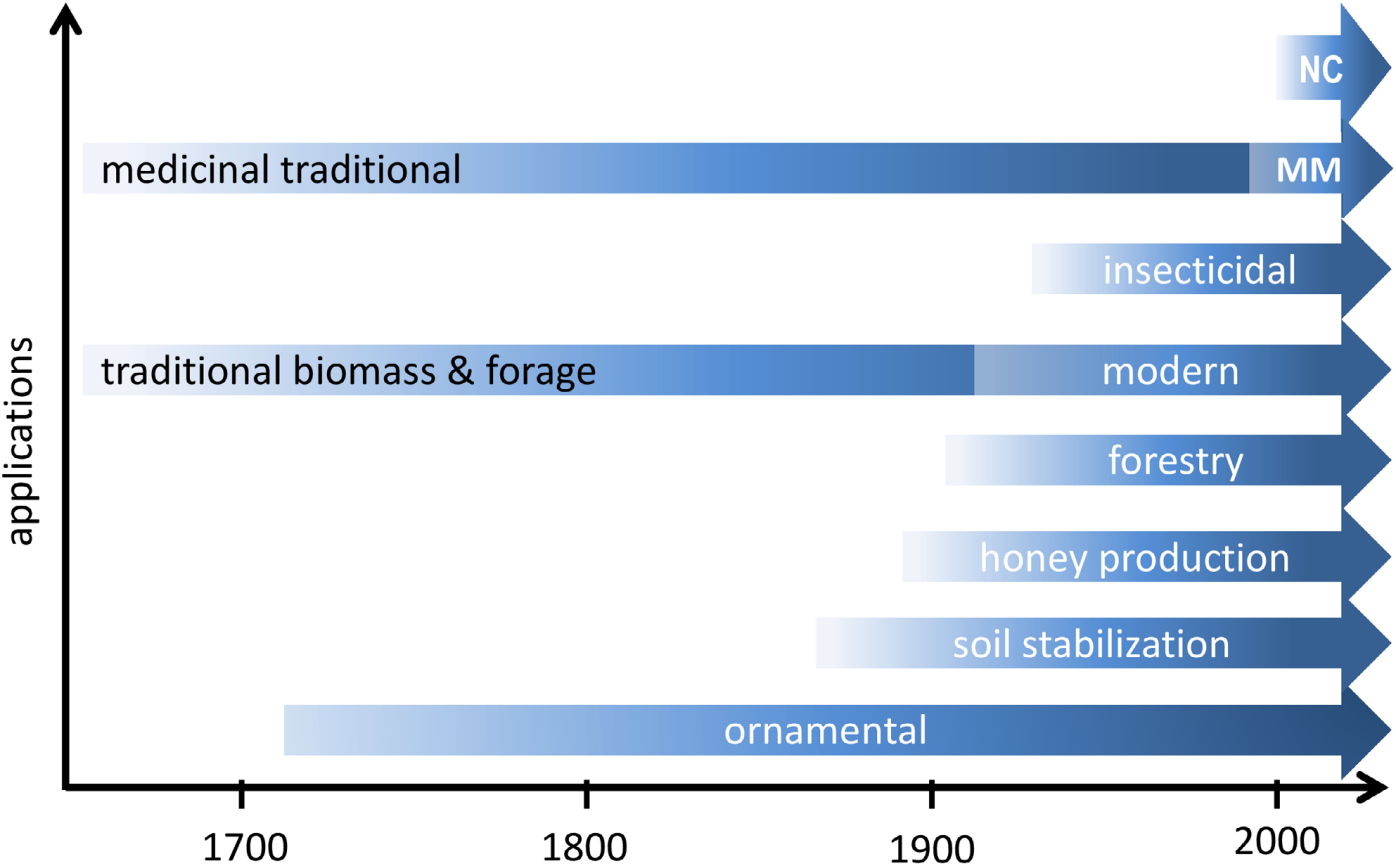
Timeline of diversifying *Amorpha fruticosa* applications

During the centuries, the species has been used traditionally for forage, woody biomass, indigenous medicine etc. Still, lately, its applications have been diversified, and sophisticated technologies were applied for some uses, for example, for research in the field of medicine or for obtaining nanocellulose (Figure 7). Currently, there are many applications of *A. fruticosa* for beneficial purposes, which can be divided into three groups: (a) Phytochemistry: use of plant compounds as pharmaceuticals, pesticides, and biomaterials; (b) Biocoenotic uses: honey plant, ornamental purposes, and (c) Ecosystem services: forage and biomass; forestry and soil erosion prevention, phytoremediation, etc. While no products currently use this species, efforts have been made to research its pharmaceutical uses, indicating the potential that it could be used as a base raw material for a variety of applications. Detailed elaboration of each group is given in the text that follows.

#### 7.1.1 Application of *A. fruticosa* in phytochemistry

There is a wide spectrum of *A. fruticosa* applications for medicinal purposes intended to treat symptoms and diseases where the plant has been proven effective. In traditional medicine the plant leaves have been used, since they are slightly bitter, inducing cooling effect. Numerous compounds contained in *A. fruticosa* are showing medical properties and in this review, we will present just a few examples. In modern medicine, application have been diversified mainly due to beneficial properties of rotenoids and isoflavones. Therefore, *A. fruticosa* is used for stimulating immunity, treating diabetes, metabolic disease, and cancer (Cvetković et al., 2019; Kozuharova et al., 2017; Lee et al., 2006; Lee et al., 2016), as well as possessing antimicrobial properties and for treating stomach pain, intestinal worms, eczema, neuralgia, carbuncle, burns, wounds, and rheumatism. In search for possible antitumor agents, Li et al. (1993) extracted 8 novel cytotoxic compounds, belonging to rotenoids and isoflavones. Furthermore, Muharini et al. (2017) found 14 new natural compounds, together with 40 already known isolated from the fruits of *A. fruticosa*, and tested them for their antimicrobial activity. Some compounds showed potent to moderate antibacterial activities against several Gram‐positive bacteria. The same research confirmed that some natural compounds derived from fruits of *A. fruticosa* were significantly cytotoxic against the mouse lymphoma cell line. In Table 2 are summarized medical properties of the plant, concerning traditional use and new applications in medicine. Additionally, an extensive review of *A. fruticosa* medical properties is given by Kozuharova et al. (2017).

**TABLE 2:** Summary of medical and pharmaceutical properties of *Amorpha fruticosa*

Potential medical uses/disease	Plant part	Proven to be effective	References
Antidiabetic properties	Fruit		Weidner et al. (2012), Lee et al. (2016)
Stomach pain, intestinal worms, eczema, neuralgia	Leaves	Traditional use	Hoffman (1891), Gilmore (1919), Smith (1928), Straub (2010)
Rheumatism	Leaves and stems	Traditional use	Munson (1981), Austin (2004)
Wounds		Traditional use	Munson (1981), Austin (2004)
Antitumor agents: cytostatic or cytotoxic	Fruit	Isolation of 8 cytotoxic constituents, rotenoids, and isoflavones	Li et al. (1993), Zhu et al. (2017)
	Fruit	Significantly cytotoxic against the L5178Y mouse lymphoma cell line	Muharini et al. (2017)
	Leaf	Retenoids have shown significant anti‐tumor effect on mouse skin tumor	Konoshima et al. (1993)
Stimulating immunity	Fruit	Stimulating growth of human T cells	Lee et al. (2006)
Antimicrobial	Fruit, leaf, root	Positive antimicrobial effect on certain Gram positive and Gram negative bacteria	Mitscher et al. (1981), Hovanet et al. (2015), Muharini et al. (2017), Kim et al. (2011)

Another potential beneficial use is in obtaining insecticidal formulations. Historically, already during the first half of the 20th century, Brett (1946) investigated *A. fruticosa* for its insecticidal properties. Tests on 29 species of insects and mites showed that the extract acted as a stomach and contact poison for tested species. In addition, it showed repellent ability to house and horn flies for more than 12 h when sprayed on cattle. The most susceptible to it were chinch bugs, cotton aphids, pea aphids, chrysanthemum aphids, and spotted cucumber beetles. According to research by Qu et al. (1998), 4 active compounds from leaves of *A. fruticosa* were isolated: (1) 6alpha, 12alpha‐dehydro‐alpha‐toxicorol, (2) 6alpha, 12alpha‐dehydro‐deguelin, (3) (±)‐tephrosin, and (4) (−)‐6‐hydroxy‐6alpha. 12alpha‐dehydro‐toxicarol. These compounds are nontoxic and are safe and reliable for humans and livestock. The results showed that none of these compounds had an insecticidal effect when administered alone but showed an ideal insecticidal effect when mixed in a certain proportion. The results indicate that using *A. fruticosa* leaves is very promising as a source of biological pesticide. Liang et al. (2015) have investigated the influence of amorphigenin (8′‐hydroxyrotenone), a rotenoid compound, isolated from the seeds of *A. fruticosa* on larvae of the mosquito *Culex pipiens pallens* (Diptera: Culicidae). Isolated amorphigenin exhibited a strong larvicidal activity with LC_50_ and LC_90_ values of 4.29 and 11.27 mg/L, respectively. Mingshan et al. (2015) proved the same thing, explaining that amorphigenin effectively inhibits the activity of the mitochondrial complex I.

Finally, one of the emerging uses of *A. frutiosa* is processing its tree biomass to obtain novel biomaterial—nanocellulose. Nanocellulose is characterized by biocompatibility, biodegradability, high mechanical strength, abundant hydroxyl groups for potential functionality, and above all, it is a renewable material. Zhuo et al. (2017) managed to isolate nanocellulose from *A. fruticosa* by applying a low energy input method for extraction. Such performances of produced nanocellulose could be further applied in various domains like electronics, biomedicine, and aerospace.

#### 7.1.2 Biocoenotic uses and ecosystem properties

It has already been elaborated that *A. fruticosa* can have a suppressive effect on native biodiversity due to its ability to reproduce and adapt successfully. Nevertheless, its reproduction is facilitated by pollinators which are in turn attracted by numerous inflorescences rich in pollen and nectar. *Amorpha fruticosa* represents an important food source for bees and other insects within its native and new habitats where it has been introduced (Kozuharova et al., 2017). Therefore, it is regarded as a significant honey plant from the point of ecosystems and beekeepers (Li et al., 2014; Stefanic et al., 2004). In addition, since the 18th century, *A. fruticosa* has been considered a valuable decorative plant (Cullen, 1995). Its growth, phenology, adaptability, and resistance make the plant suitable for different types of urban green spaces and gardens in China. Above all, it is considered a water‐saving plant suitable for planting in urban areas with water shortages, such as in Beijing (Huang, 2005).

#### 7.1.3 Ecosystem services *A. fruticosa* provides

One of the most frequently used ecosystem services is the *A. fruticosa*'s ability to stabilize soil on slopes. It has been widely used in forestry, preventing soil erosion, and reclamation of degraded environments (DeHaan et al., 2006; Yin, 1993). There are two ways by which *A. fruticosa* can contribute to preventing soil erosion (Yin, 1993): (1) it is fast‐growing and establishes a closed canopy in a short time, covering the ground quickly and thus intercepting rainfall and preventing soil erosion, and (2) mature *A. fruticosa* stands produe yearly a large amount of dead plant material thus protecting soil surface from splashing erosion. Therefore, the species has been considered as an ideal tree species for protecting forests at gully head and fixing the bank and cliff of slope embankment, soil consolidation, slope protection, ditch protection, as well as on railway embankments, and soil and water conservation (Kozuharova et al., 2017; Yin, 1993). In addition, it is also considered as suitable for revegetation of moderately saline soils (Đukić et al., 2010; Guo et al., 2018) and for stabilizing metals from Pb to Zn mine tailings (Sikdar et al., 2020), opening a new possibility for its application—phytomining.

*Amorpha fruticosa* expresses negative allelopathic effect toward some plant species (Xiao et al., 2016), while stimulating some others (Wang et al., 2018). Interestingly, the growth of another invasive plant *Phytolacca americana* could be controlled at the sites where *A. fruticosa* was in vigorous growth (Fu et al., 2012). Besides, there are positive examples where *A. fruticosa* has been used as intercropping plant (Lygis et al., 2004), and possibilities to use it as for forage and biomass (DeHaan et al., 2006). Table 3 summarizes applications of *A. fruticosa* apart from medical purposes.

**TABLE 3:** Usage summary of *A. fruticosa*, apart from medical purposes

Plant part	Usage	References
Flowers	Honey plant	Stefanic et al. (2004), Li et al. (2014), Kozuharova et al. (2017), Zhu et al. (2020).
Seeds	Insecticidal—amorhinogenin to larvae of the mosquito *Culex pipiens pallens* (Diptera: Culicidae)	Liang et al. (2015)
	Insecticidal, repellent	Brett (1946), Qu et al. (1998)
Fruit	Amorfrutins used as an ingredient in some condiments	Kozuharova et al. (2017)
Branches	Weaving of baskets and fences	Traditional use
Whole plant/roots	Forestry—soil stabilization, erosion prevention, windbreak, shelterbelt	Yin (1993), DeHaan et al. (2006), Wang et al. (2011), Xiaolei et al. (2016), Kozuharova et al. (2017), USDA (2019)
	CO_2_ sequestration, enriching soil with nitrogen	Ciuvăţ et al. (2016)
Whole plant	Ornamental plant—pot and garden	Cullen (1995), Huang (2005), Kozuharova et al. (2017)
Whole plant/woody biomass	Biomass energy	DeHaan et al. (2006), Guo et al. (2018)
	Nanocellulose	Zhuo et al. (2017)
	Cellulose, pellet	Ciuvăţ et al. (2016)
Leaf material	Livestock forage	DeHaan et al. (2006), Guo et al. (2018)
	Green manure	DeHaan et al. (2006)

### 7.2 Amorpha fruticosa spreading control: Costly or sustainable?

**FIGURE 8:**
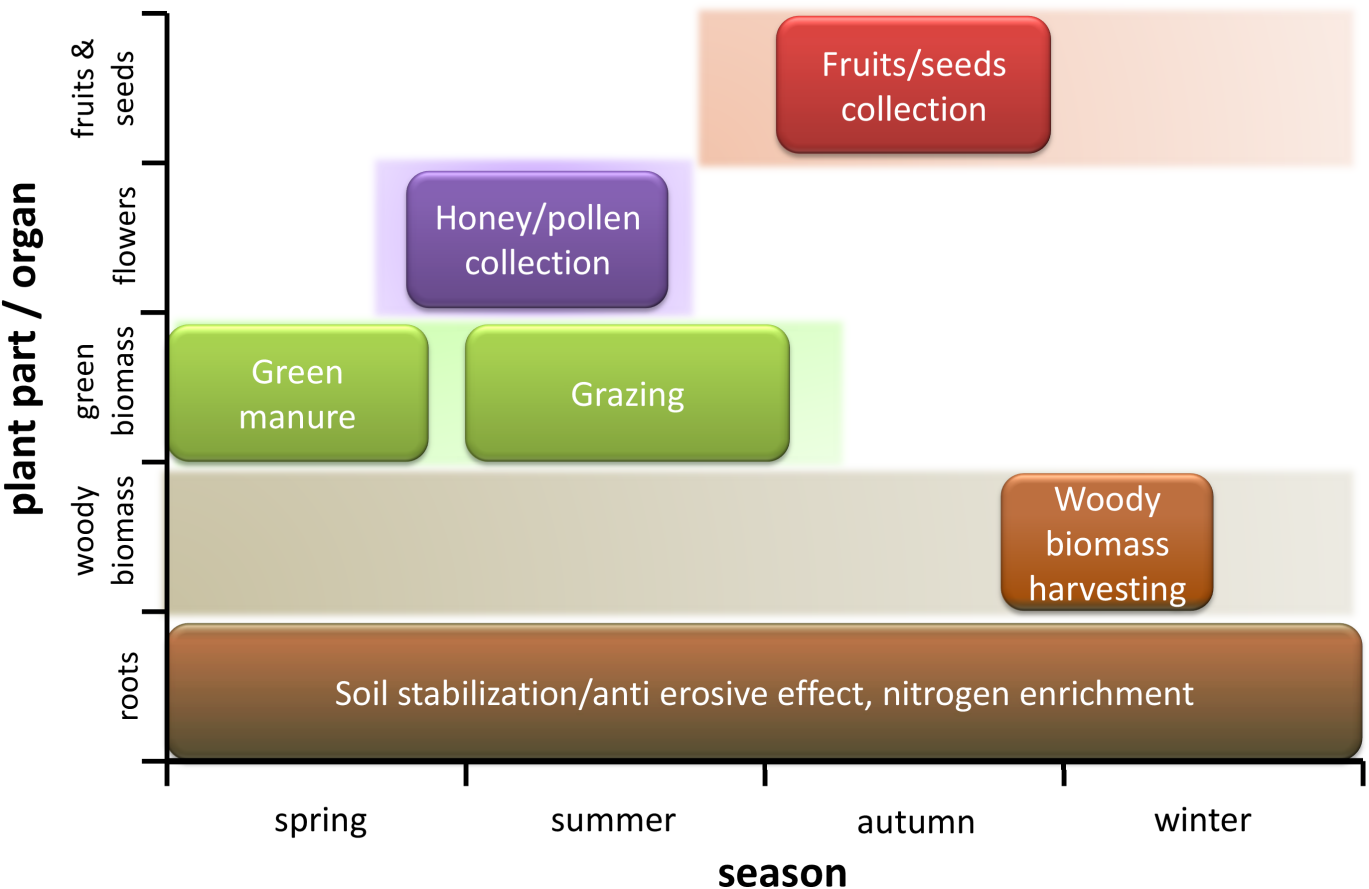
Possible utilization of different organs of *Amorpha fruticosa* throughout the year

Active measures to control *A. fruticosa* spreading are often labor‐intensive and costly, but valuating its biological potential and finding an economically viable solution might represent a sound approach. Therefore, Ciuvăţ et al. (2016) propose a three‐stage utilization of *A. fruticosa* covering the whole year: the 1st stage in spring/summer —honey and pollen collecting by bees; the 2nd stage in autumn —seeds collecting for medical/pharmaceutical purposes and the 3rd stage in late autumn/winter —harvesting woody biomass as raw material for industry. In addition, leaves and green parts can be used for feeding cattle and game animals throughout the vegetation season, or as green manure (DeHaan et al., 2006). Nevertheless, throughout the year, roots contribute to soil stabilization and nitrogen‐enrichment (Figure 8). So far, no study on the economic aspect of exploitation benefits vs expenses for spreading control of *A. fruticosa* was published. One of the important aspects is collecting *A. fruticosa* for raw material, which is often considered costly. However, the fact is that when an area gets overgrown by *A. fruticosa*, it forms impenetrable stands, almost monocultures, which facilitate its collecting. Among numerous possibilities for exploiting *A. fruticosa*, it seems that the most interesting is that its extract has repellent abilities against mosquitoes and other pests. The fact is that the species inhabits wetlands where also mosquitoes are abundant. Making a repellent product out of locally harvested *A. fruticosa* would also encourage tourism and thus benefit the economy of the protected area managing *A. fruticosa*. Nevertheless, the issue of financial compensation for controlling *A. fruticosa* and its beneficial products needs further examination.

Additionally, applied measures have to be chosen by the importance of the area, for example, within a protected area invasive species has to be completely eradicated, but only utilizing allowed management practices. In other areas, depending on the degree of invasion and naturalization, the application of different measures and/or their alternations may be justified. Otherwise, in areas where introduced tree species has heavily modified the ecosystem, where restoration is not possible or even undesirable to some historical condition, society has to accept changes and learn how to live with the invasive species and alterations it caused (Richardson et al., 2014). In such a context can be viewed *A. fruticosa* profound invasion of riparian areas along watercourses—forests, wet meadows, or poplar plantations. The choice of measures will be dependent on the goal which needs to be achieved, concerning available labor and financial resources, or some other means indirectly related to humans, such as establishing grazing practice or releasing agents of biological control. For example, the best results of control in floodplain meadows and poplar plantations affected by *A. fruticosa* are achieved by applying continuous to moderate or intensive cattle grazing. This measure not only contributes to successful suppression of the invasive plant, but also enhances local biodiversity, reduces flood risk, helps in developing local communities by providing additional grazing areas that is, maintaining traditional land‐use practices (Demeter et al., 2021) and even helping manage poplar and other soft‐wood plantations. Therefore, this management option is truly multifunctional leading a “win–win–win” scenario (Demeter et al., 2021). On the contrary, in an area where a lower level of invasion is recorded, control of invading species is desirable and achievable. Nevertheless, the management of an invasive species is quite complex and it requires transdisciplinary endeavors (Richardson et al., 2014). This is especially necessary when a species shows dualistic nature—negative toward the environment and simultaneously satisfying human needs. Such challenging tasks require multidimensional evaluation including an interdisciplinary team which takes into consideration ethics, law, policy, ecology, and natural resources management (Schwartz et al., 2012), leading to the development of pragmatic solutions and innovative approaches to conflict resolution (Hobbs et al., 2013; Richardson et al., 2014).

## 8 CONCLUSION

The plant species *A. fruticosa* is quite controversial. In areas where it has been intentionally spread, especially for soil stabilization along canals, it represents a real threat to native biodiversity. There are a variety of measures that have proven to be effective for its spread control (mechanical, chemical, and biological, or alternating their application). Still, these are often labor demanding and costly, except when grazing is applied. On the other hand, the plant is quite beneficial for many purposes, such as: in forestry for erosion control, ornamental/decorative purposes, being honey plant, potential source of medical compounds, insecticidal properties, source of animal feed, fibre and woody biomass, etc. Therefore, *A. fruticosa* should not be introduced to new locations without a detailed risk assessment. However, in historically heavily affected regions a win‐win scenario would be promoting its use for beneficial purposes, thus achieving speeding control in a cost‐effective and sustainable manner.

Future perspectives may be oriented in two directions: investigation of proper integrated management strategies on spreading control, adequate for certain regions, and further exploration of beneficial effects. In both cases, finding economic value for *A. fruticosa* uses may help. Therefore, smart management in invaded areas based on its controlled exploitation for beneficial purposes could be a leading strategy in future *A. fruticosa* spreading control.

## AUTHOR CONTRIBUTIONS

**Jasna Grabic:** Conceptualization (lead); data curation (lead); funding acquisition (lead); project administration (lead); writing – original draft (lead); writing – review and editing (equal). **Branka Ljevnaić‐Mašić:** Conceptualization (equal); data curation (equal); validation (equal); visualization (equal); writing – original draft (equal). **Ai Zhan:** Data curation (equal); writing – review and editing (equal). **Pavel Benka:** Formal analysis (lead); software (lead); visualization (equal); writing – review and editing (equal). **Hermann Heilmeier:** Methodology (equal); supervision (lead); validation (equal); visualization (equal); writing – review and editing (equal).

## CONFLICT OF INTEREST

None declared.

## Data Availability

The datasets on the frequency of *A. fruticosa* in literature were generated from Web of Science—WOS [https://www.webofscience.com/woscc/basic‐search], PubMed [https://pubmed.ncbi.nlm.nih.gov/], ScienceDirect [https://www.sciencedirect.com/], and Scopus [https://www.scopus.com/home.uri] repository, while data on distribution of the same species were obtained from GBIF [https://www.gbif.org/species/5357407].
